# Correction: Combined SPT and FCS methods reveal a mechanism of RNAP II oversampling in cell nuclei

**DOI:** 10.1038/s41598-025-26581-1

**Published:** 2025-11-18

**Authors:** Marie Fournier, Pierre Leclerc, Aymeric Leray, Dorian Champelovier, Florence Agbazahou, Fatima Dahmani, Gabriel Bidaux, Alessandro Furlan, Laurent Héliot

**Affiliations:** 1 Univ. Lille, CNRS, UMR 8523, PhLAM Laboratoire de Physique des Lasers, Atomes et Molécules, Lille, France; 2 Laboratoire Interdisciplinaire Carnot de Bourgogne, UMR 6303 CNRS, Université de Bourgogne Franche Comte, Dijon, France; 3 INSERM UMR 1060, CarMeN Laboratory, IHU OPERA, Hôpital Louis Pradel, Hospices Civils de Lyon, Univ Lyon1, Lyon, France; 4 Univ. Lille, CNRS, Inserm, CHU Lille, UMR9020-U1277 -CANTHER -Cancer Heterogeneity Plasticity and Resistance to Therapies, Lille, 59000 France; 5 Unité Tumorigenèse et Résistance aux Traitements, Centre Oscar Lambret, 59000 Lille, France; 6 CNRS, Groupement de Recherche ImaBio, 59655 Villeneuve d’Ascq, France

Correction to: *Scientific Reports* 10.1038/s41598-023-38668-8, published online 05 September 2023

The original version of this Article contained errors.

In the Results section, under the subheading ‘Characterization of calibrated bead diffusion by ACF analysis of FCS measurements’

“Up to a glycerol content of 50% in water, the AutoCorrelation Function (ACF) analysis yielded an anomalous coefficient value very close to the theoretical value of 1 (between 0.96 and 1.02), as expected for Brownian diffusion (Fig. 1A). Only the diffusion of beads in the solution containing 80% glycerol was described as anomalous, with an alpha of 0.70 ± 0.02.”

now reads:

“Up to a glycerol content of 50% in water, the AutoCorrelation Function (ACF) analysis yielded an anomalous coefficient value very close to the theoretical value of 1 (between 0.90 and 1.07), as expected for Brownian diffusion (Fig. 1A). Only the diffusion of beads in the solution containing 80% glycerol was described as anomalous, with an alpha of 0.81.”

Under the subheading ‘Characterization of calibrated bead diffusion by SPT analysis’,

“As observed in FCS, a high content of glycerol (80%) resulted in an alpha compute/d at 0.8, with this apparent anomaly probably associated with a certain heterogeneity of the mixture.”

now reads:

“As observed in FCS, a high content of glycerol (80%) resulted in an alpha computed at 0.72, with this apparent anomaly probably associated with a certain heterogeneity of the mixture.”

In addition, Figure 2A was inadvertently identical to Figure 1A. The original Figure 2 and accompanying legend appear below.

**Fig. 2:**
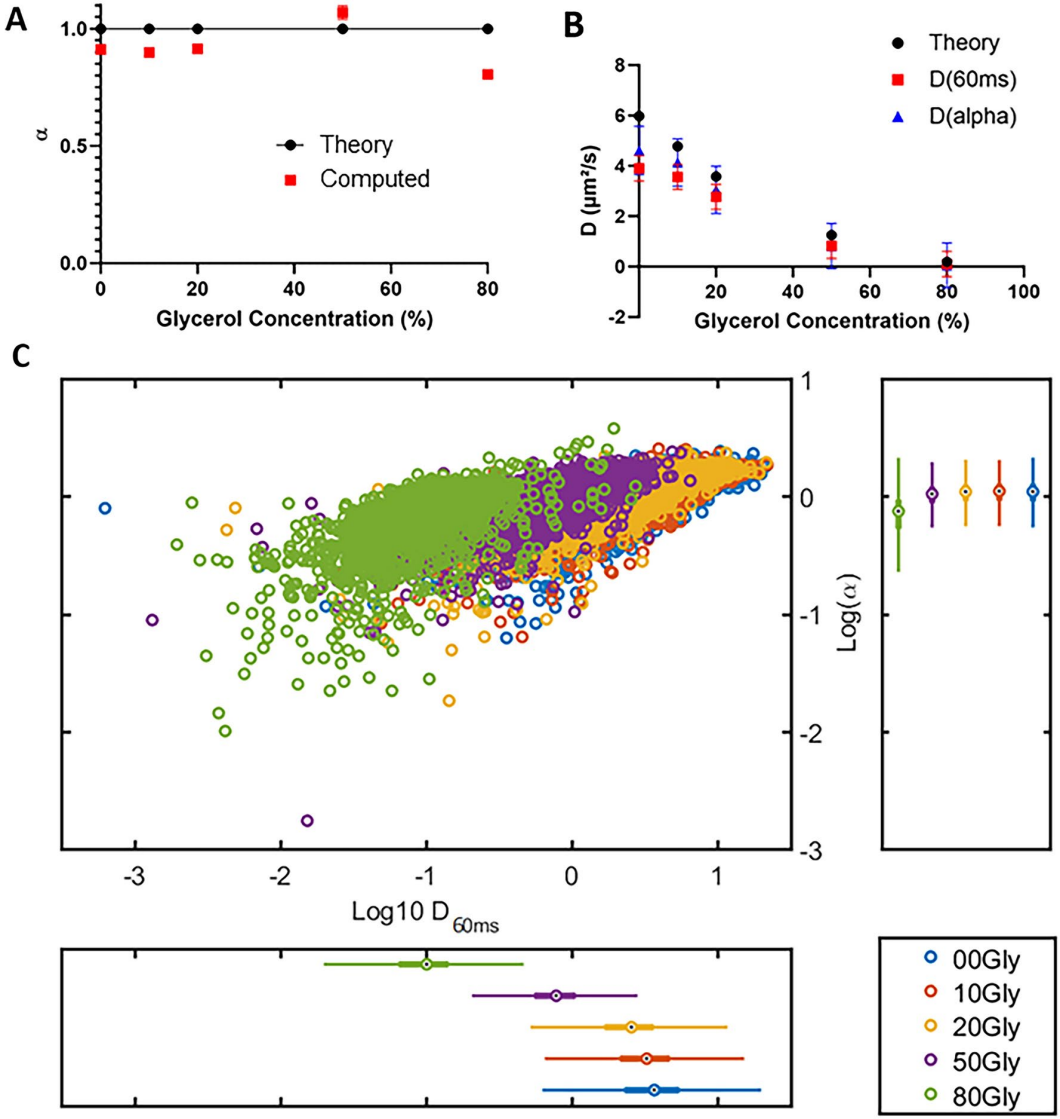
Characterization of diffusion by Single Particle Tracking. Fluorescent microspheres were resuspended in water/glycerol mixtures, with proportions of glycerol ranging from 0 to 80%. SPT acquisitions were performed at a frame rate of 100 Hz. (**A**) The anomalous coefficient α is provided as a function of the glycerol proportion. Experimental values (red squares) can be compared to theoretical values (black dots). (**B**) The diffusion coefficients were computed by two methods: D_a_ (blue triangles) were obtained from the slopes of TEA-MSD (Time Ensemble Average Mean Square Displacement) expressed as log, while D_60ms_ were calculated for each trajectory during the first 60 ms of its occurrence (red squares). These experimental values could be compared to theoretical values (black dots). Both methods yielded an asymptotic trend for high D values. (**C**) The distributions of D_60ms_ and α from the different experimental conditions are represented with log scales in a scatter plot. Distinct subpopulations can be distinguished (green, purple, and yellow dots) within a limited range of parameters, whereas some others overlap (yellow, red, and blue dots). Data come from at least one hundred measurements per condition.

Furthermore, Table 1 contained erroneous values. The original Table 1 and accompanying legend appear below.

**Table 1:** Diffusion coefficient values of glycerol in water solutions, at different ratios, calculated following^28^.

% Glycerol	Calculated D value (µm^2^/s) for 40 nm beads	Calculated D value (µm^2^/s) for 100 nm beads
0	15.1	5.98
10	12.1	4.78
20	9.05	3.58
50	1.25	1.25
80	0.19	0.19

Finally, Supplementary Table 1 contained erroneous values. The original Supplementary Table 1 and accompanying legend appear below.

**Supplementary Table 1:** Comparison of experimental vs theoretical values of anomaly coefficients (A) and diffusion coefficients (B) computed from the ACF of FCS measurements performed with fluorescent beads diffusing in aqueous solutions with different glycerol proportions.

(A) Anomaly coefficient	Experimental values		Theory
% glycerol	mean α	SD	α
0	0.96	0.01	1
10	0.94	0.02	1
20	0.97	0.02	1
50	1.02	0.02	1
80	0.7	0.02	1

(B) Diffusion coefficient	Experimental values		Theory
% glycerol	mean ${D}_{\alpha }$ (µm^2^/s)	SD	D (µm^2^/s)
0	20,0	1.7	15.1
10	12.7	1.8	12.1
20	10.3	1.5	9.0
50	2.7	0.8	1.2
80	0.2	0.2	0.2

The original Article and accompanying Supplementary information file have been corrected.

