# Supplementary material for: Combined SPT and FCS methods reveal a mechanism of RNAP II oversampling in cell nuclei
Source: Sci Rep. 2023 Sep 5;13:14633. doi: 10.1038/s41598-023-38668-8 (PMC10480184; doi:10.1038/s41598-023-38668-8)
Supplement: Supplementary file 6 — Supplementary Figures. [file 41598_2023_38668_MOESM6_ESM.pptx]

## Slide 1
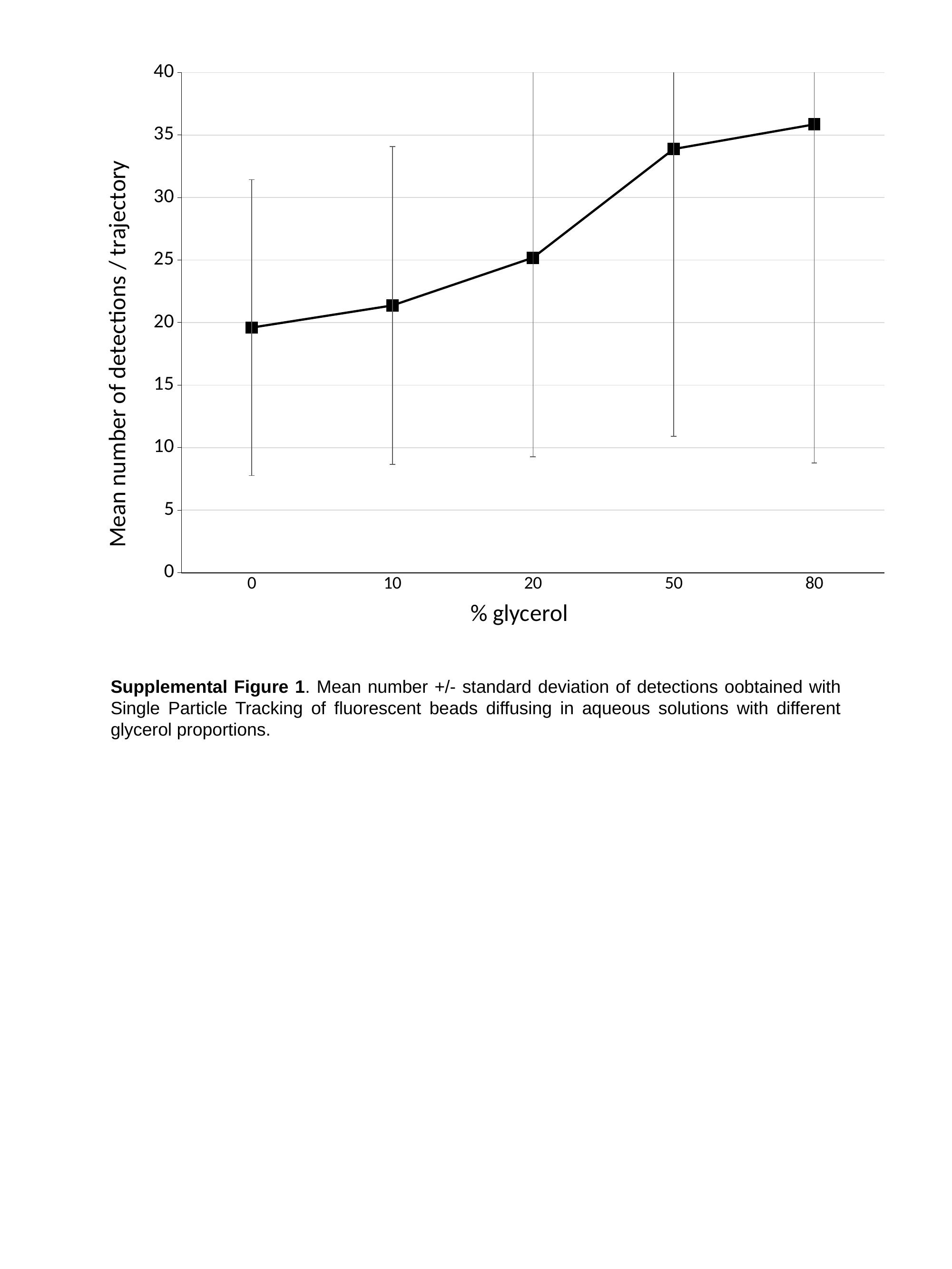

### Chart
| Category | mean |
|---|---|
| 0 | 19.6 |
| 10 | 21.37 |
| 20 | 25.19 |
| 50 | 33.88 |
| 80 | 35.85 |Supplemental Figure 1. Mean number +/- standard deviation of detections oobtained with Single Particle Tracking of fluorescent beads diffusing in aqueous solutions with different glycerol proportions.

## Slide 2
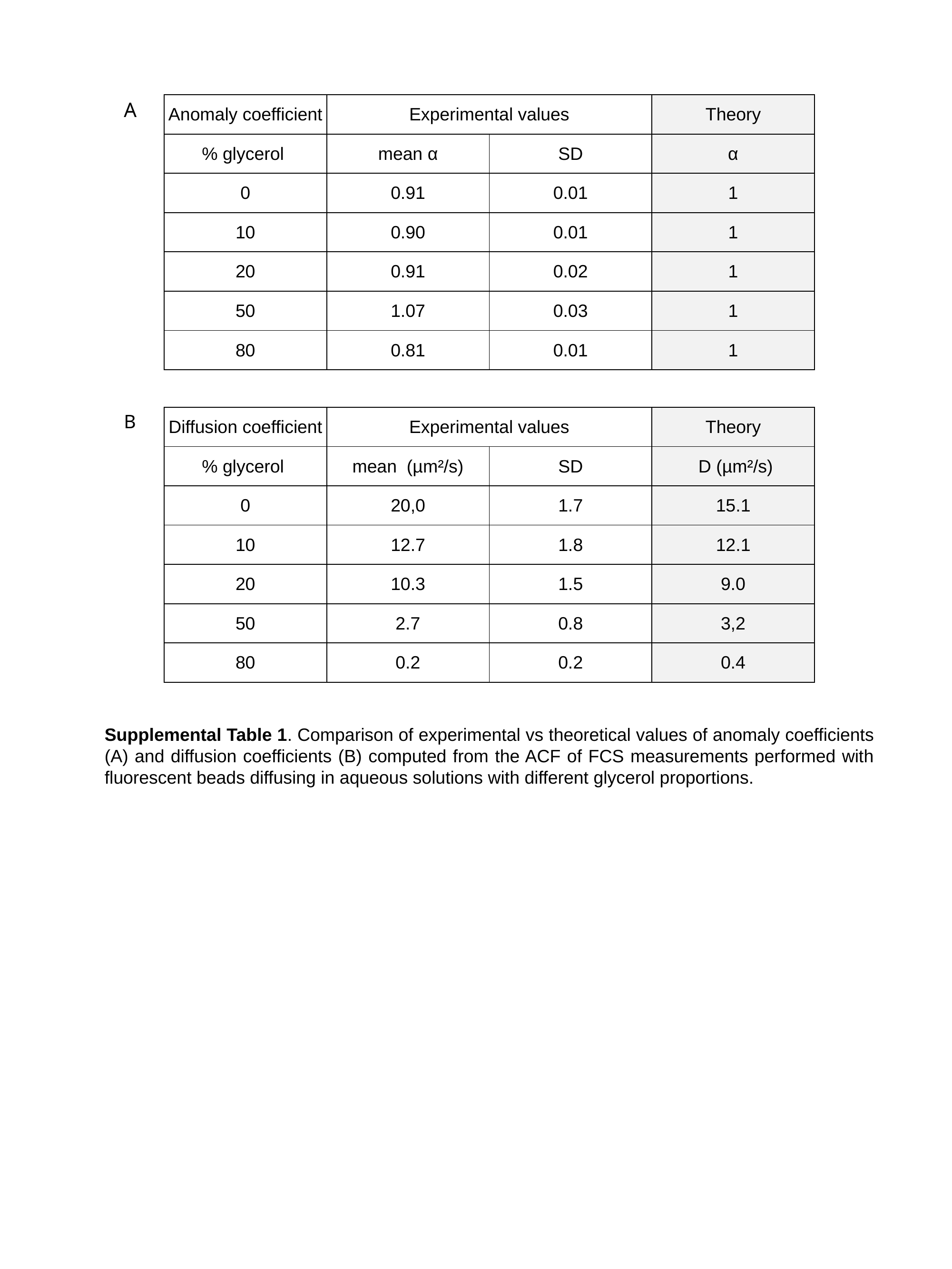

A
| Anomaly coefficient | Experimental values | | Theory |
| --- | --- | --- | --- |
| % glycerol | mean α | SD | α |
| 0 | 0.91 | 0.01 | 1 |
| 10 | 0.90 | 0.01 | 1 |
| 20 | 0.91 | 0.02 | 1 |
| 50 | 1.07 | 0.03 | 1 |
| 80 | 0.81 | 0.01 | 1 |
B
Supplemental Table 1. Comparison of experimental vs theoretical values of anomaly coefficients (A) and diffusion coefficients (B) computed from the ACF of FCS measurements performed with fluorescent beads diffusing in aqueous solutions with different glycerol proportions.

## Slide 3
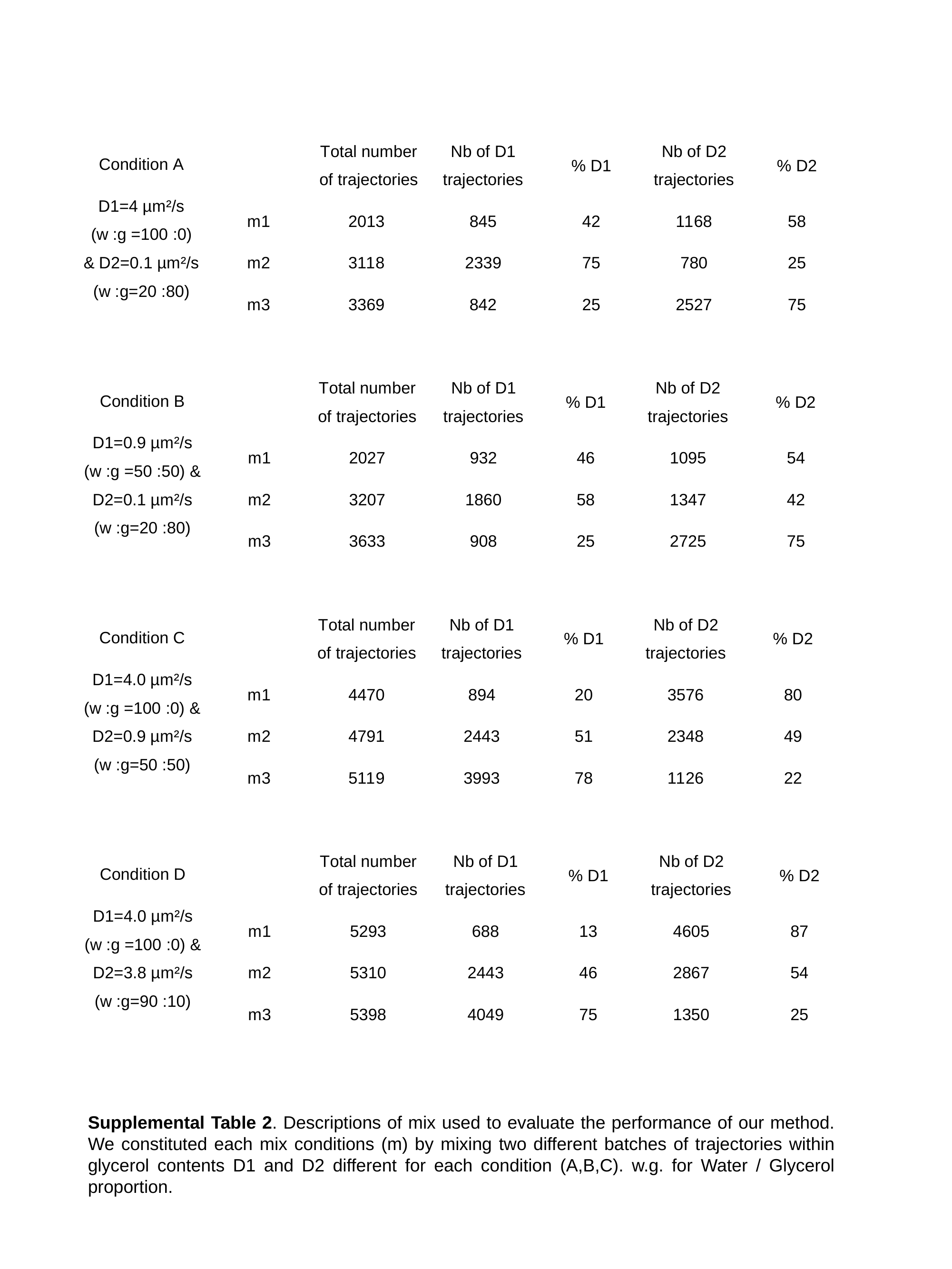

Supplemental Table 2. Descriptions of mix used to evaluate the performance of our method. We constituted each mix conditions (m) by mixing two different batches of trajectories within glycerol contents D1 and D2 different for each condition (A,B,C). w.g. for Water / Glycerol proportion.

## Slide 4
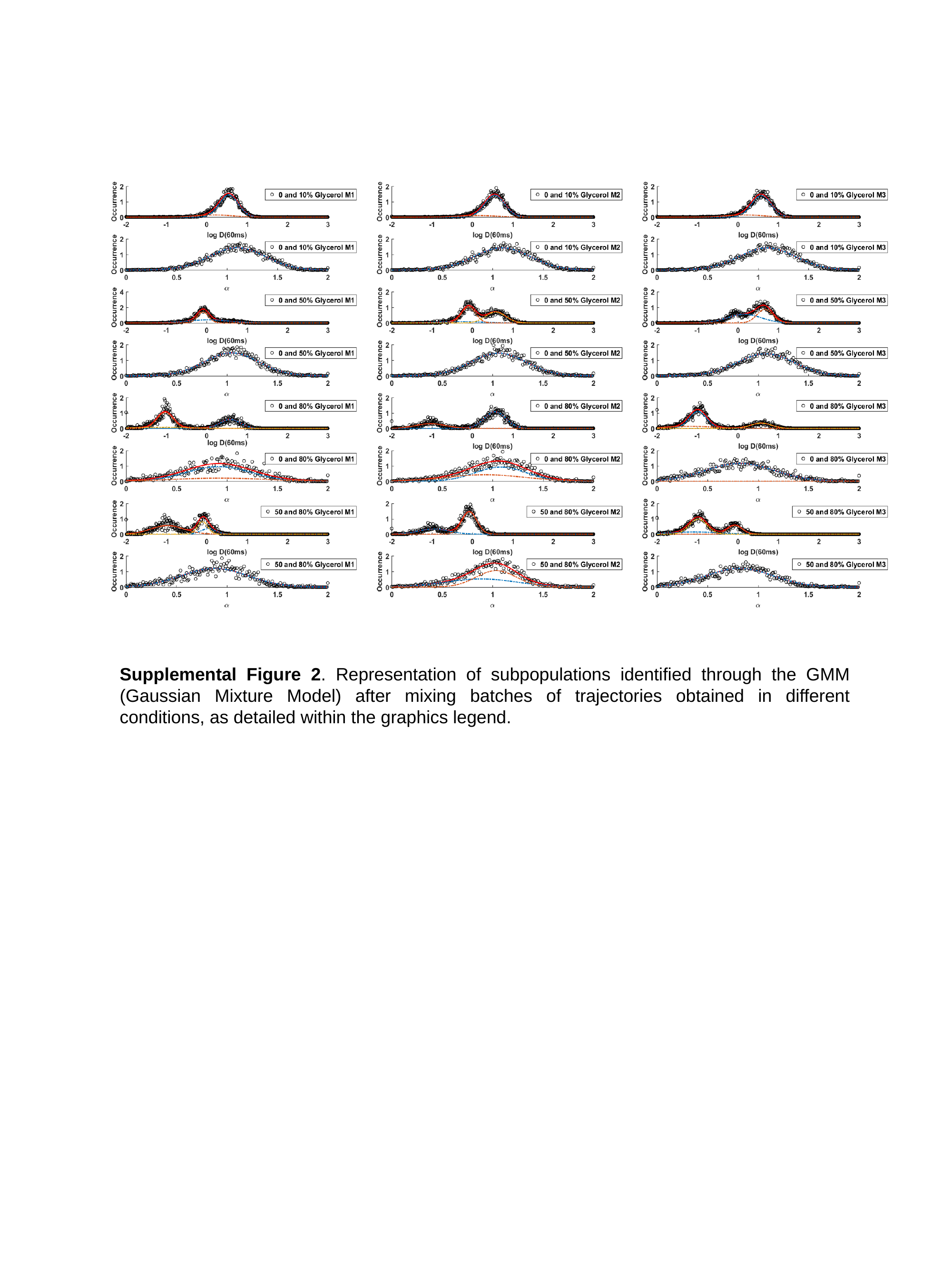

Supplemental Figure 2. Representation of subpopulations identified through the GMM (Gaussian Mixture Model) after mixing batches of trajectories obtained in different conditions, as detailed within the graphics legend.

## Slide 5
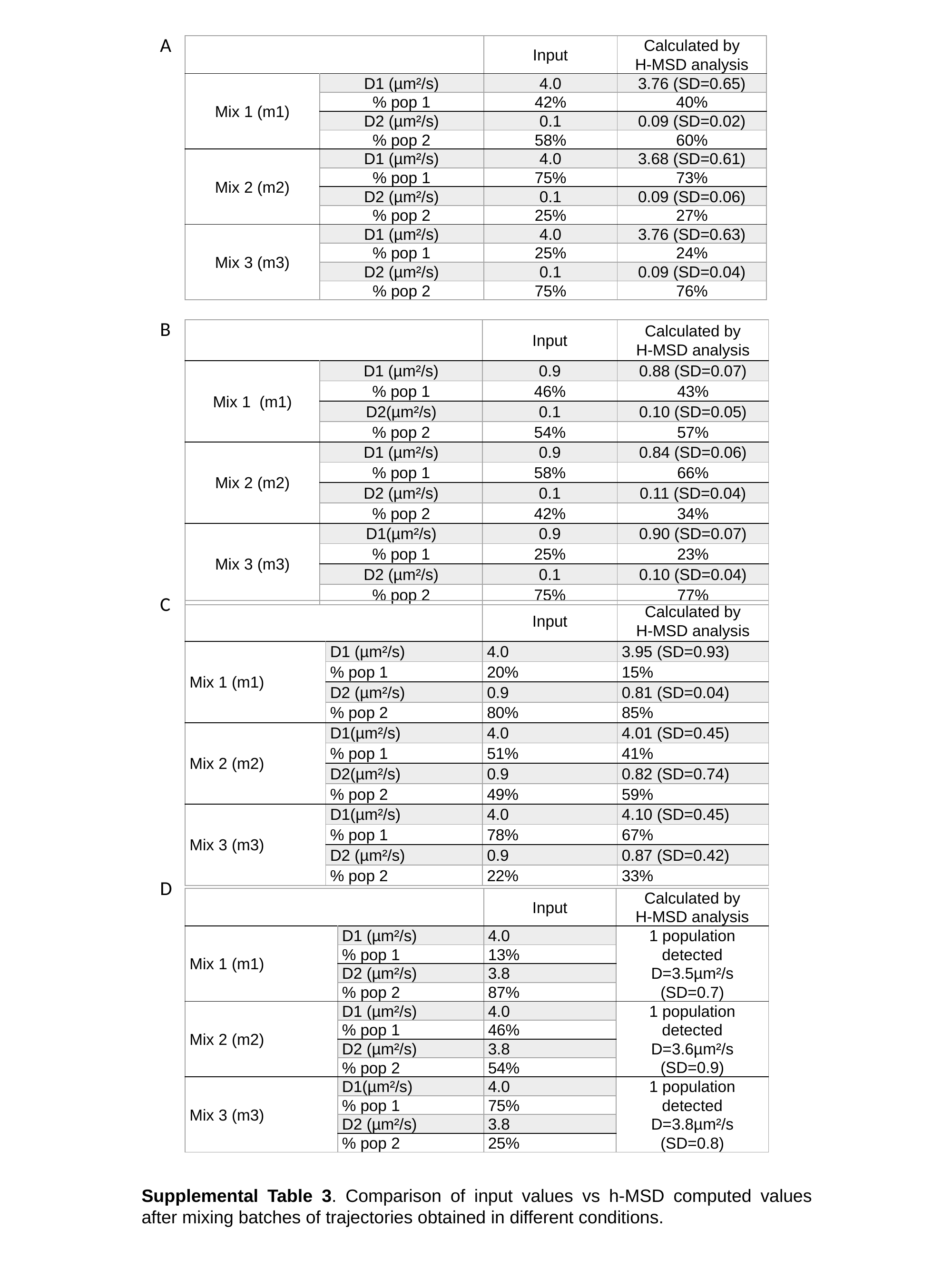

A
| | | Input | Calculated by H-MSD analysis |
| --- | --- | --- | --- |
| Mix 1 (m1) | D1 (µm²/s) | 4.0 | 3.76 (SD=0.65) |
| | % pop 1 | 42% | 40% |
| | D2 (µm²/s) | 0.1 | 0.09 (SD=0.02) |
| | % pop 2 | 58% | 60% |
| Mix 2 (m2) | D1 (µm²/s) | 4.0 | 3.68 (SD=0.61) |
| | % pop 1 | 75% | 73% |
| | D2 (µm²/s) | 0.1 | 0.09 (SD=0.06) |
| | % pop 2 | 25% | 27% |
| Mix 3 (m3) | D1 (µm²/s) | 4.0 | 3.76 (SD=0.63) |
| | % pop 1 | 25% | 24% |
| | D2 (µm²/s) | 0.1 | 0.09 (SD=0.04) |
| | % pop 2 | 75% | 76% |
B
| | | Input | Calculated by H-MSD analysis |
| --- | --- | --- | --- |
| Mix 1 (m1) | D1 (µm²/s) | 0.9 | 0.88 (SD=0.07) |
| | % pop 1 | 46% | 43% |
| | D2(µm²/s) | 0.1 | 0.10 (SD=0.05) |
| | % pop 2 | 54% | 57% |
| Mix 2 (m2) | D1 (µm²/s) | 0.9 | 0.84 (SD=0.06) |
| | % pop 1 | 58% | 66% |
| | D2 (µm²/s) | 0.1 | 0.11 (SD=0.04) |
| | % pop 2 | 42% | 34% |
| Mix 3 (m3) | D1(µm²/s) | 0.9 | 0.90 (SD=0.07) |
| | % pop 1 | 25% | 23% |
| | D2 (µm²/s) | 0.1 | 0.10 (SD=0.04) |
| | % pop 2 | 75% | 77% |
C
| | | Input | Calculated by H-MSD analysis |
| --- | --- | --- | --- |
| Mix 1 (m1) | D1 (µm²/s) | 4.0 | 3.95 (SD=0.93) |
| | % pop 1 | 20% | 15% |
| | D2 (µm²/s) | 0.9 | 0.81 (SD=0.04) |
| | % pop 2 | 80% | 85% |
| Mix 2 (m2) | D1(µm²/s) | 4.0 | 4.01 (SD=0.45) |
| | % pop 1 | 51% | 41% |
| | D2(µm²/s) | 0.9 | 0.82 (SD=0.74) |
| | % pop 2 | 49% | 59% |
| Mix 3 (m3) | D1(µm²/s) | 4.0 | 4.10 (SD=0.45) |
| | % pop 1 | 78% | 67% |
| | D2 (µm²/s) | 0.9 | 0.87 (SD=0.42) |
| | % pop 2 | 22% | 33% |
D
| | | Input | Calculated by H-MSD analysis |
| --- | --- | --- | --- |
| Mix 1 (m1) | D1 (µm²/s) | 4.0 | 1 population detected D=3.5µm²/s (SD=0.7) |
| | % pop 1 | 13% | |
| | D2 (µm²/s) | 3.8 | |
| | % pop 2 | 87% | |
| Mix 2 (m2) | D1 (µm²/s) | 4.0 | 1 population detected D=3.6µm²/s (SD=0.9) |
| | % pop 1 | 46% | |
| | D2 (µm²/s) | 3.8 | |
| | % pop 2 | 54% | |
| Mix 3 (m3) | D1(µm²/s) | 4.0 | 1 population detected D=3.8µm²/s (SD=0.8) |
| | % pop 1 | 75% | |
| | D2 (µm²/s) | 3.8 | |
| | % pop 2 | 25% | |
Supplemental Table 3. Comparison of input values vs h-MSD computed values after mixing batches of trajectories obtained in different conditions.

## Slide 6
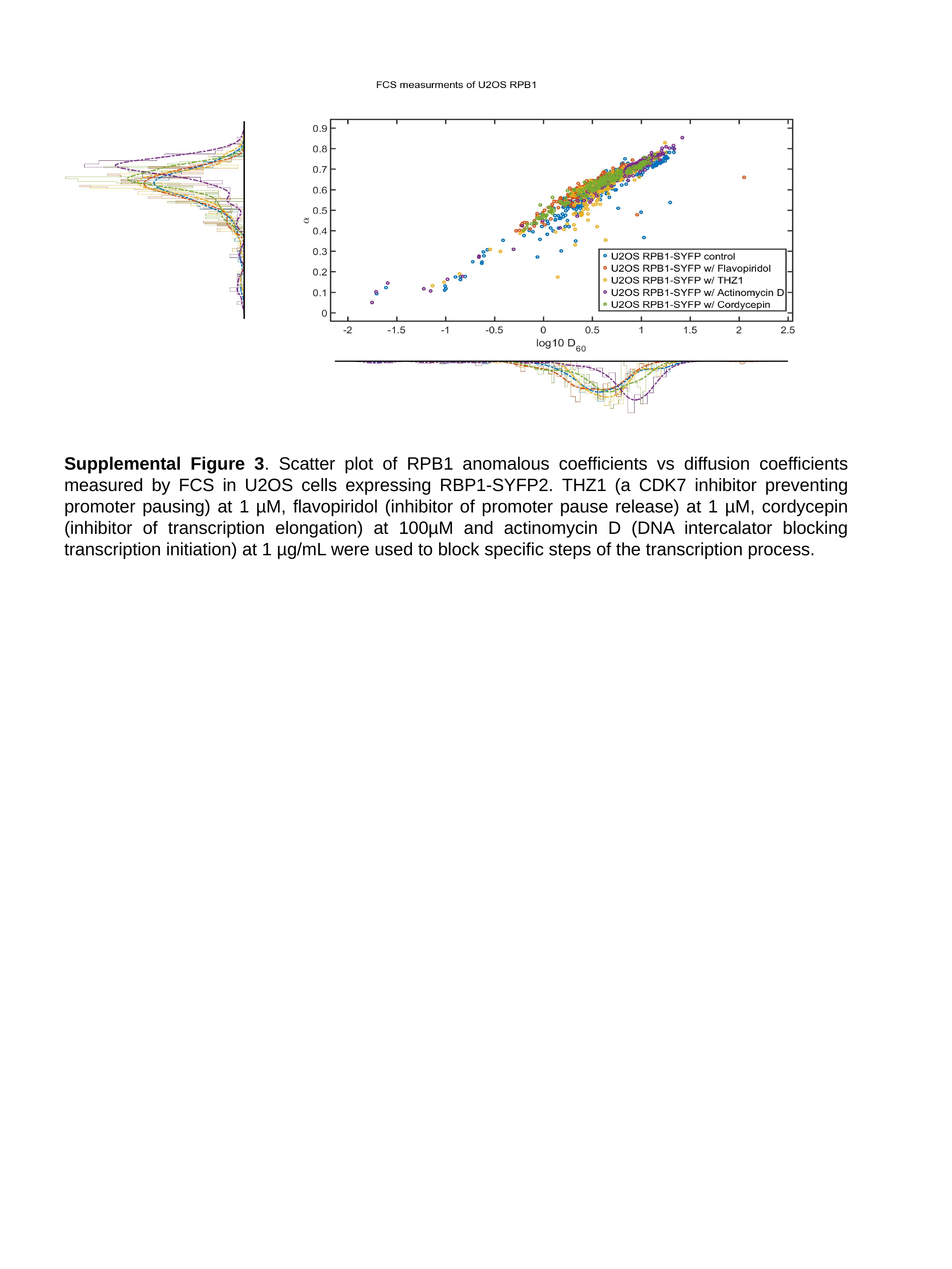

Supplemental Figure 3. Scatter plot of RPB1 anomalous coefficients vs diffusion coefficients measured by FCS in U2OS cells expressing RBP1-SYFP2. THZ1 (a CDK7 inhibitor preventing promoter pausing) at 1 µM, flavopiridol (inhibitor of promoter pause release) at 1 µM, cordycepin (inhibitor of transcription elongation) at 100µM and actinomycin D (DNA intercalator blocking transcription initiation) at 1 µg/mL were used to block specific steps of the transcription process.

## Slide 7
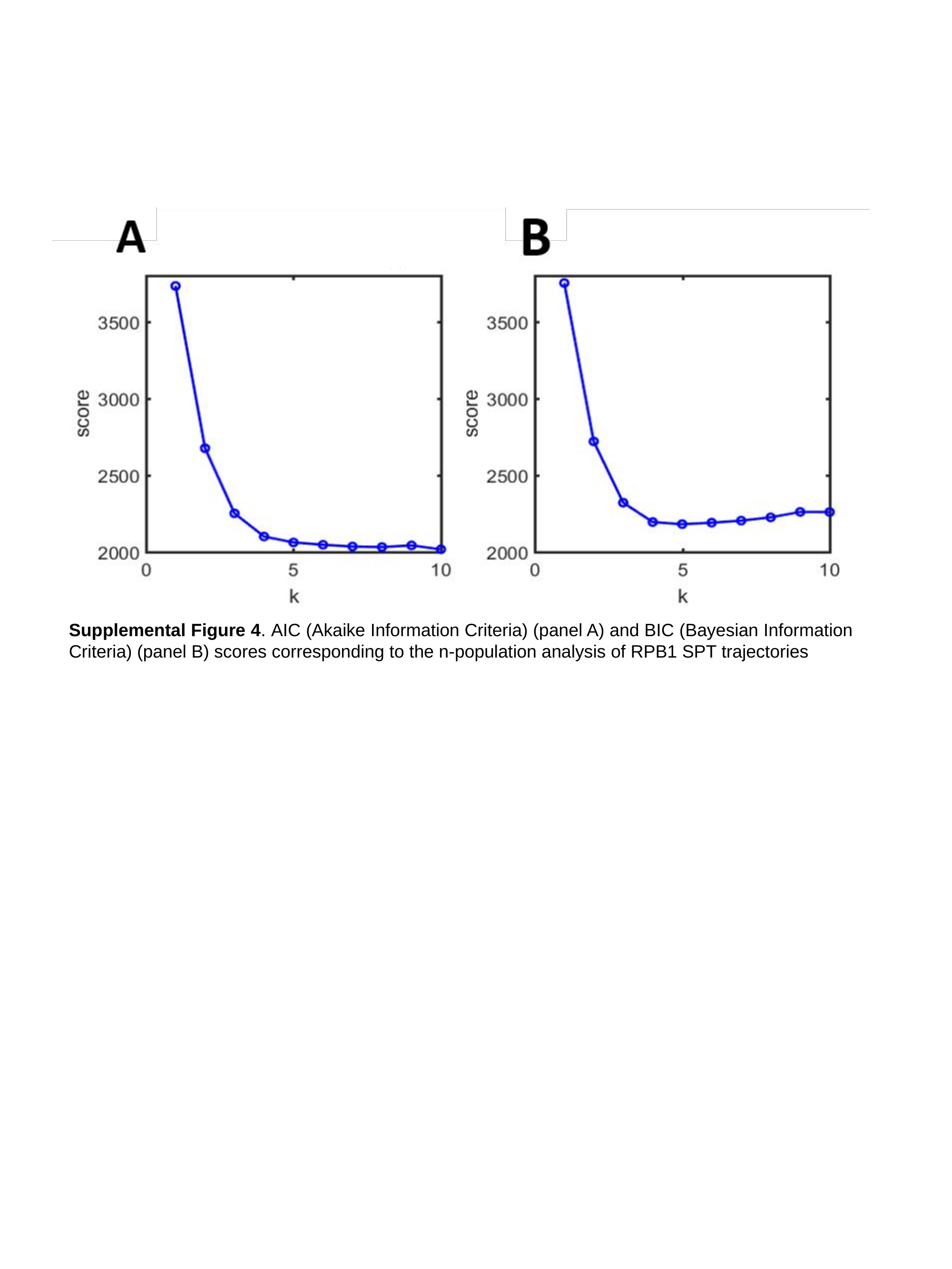

Supplemental Figure 4. AIC (Akaike Information Criteria) (panel A) and BIC (Bayesian Information Criteria) (panel B) scores corresponding to the n-population analysis of RPB1 SPT trajectories

## Slide 8
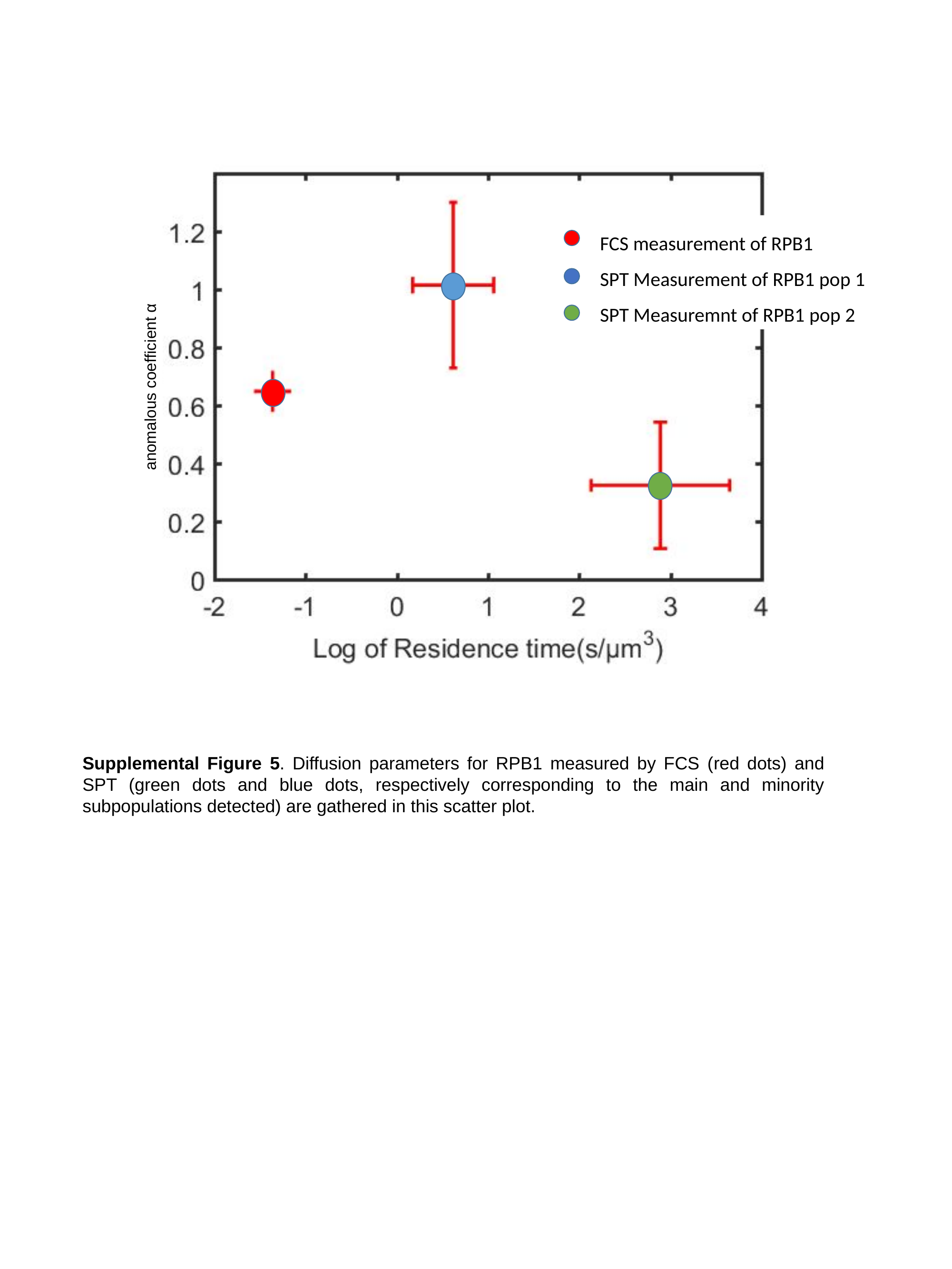

FCS measurement of RPB1
SPT Measurement of RPB1 pop 1
SPT Measuremnt of RPB1 pop 2
anomalous coefficient α
Supplemental Figure 5. Diffusion parameters for RPB1 measured by FCS (red dots) and SPT (green dots and blue dots, respectively corresponding to the main and minority subpopulations detected) are gathered in this scatter plot.
